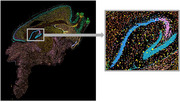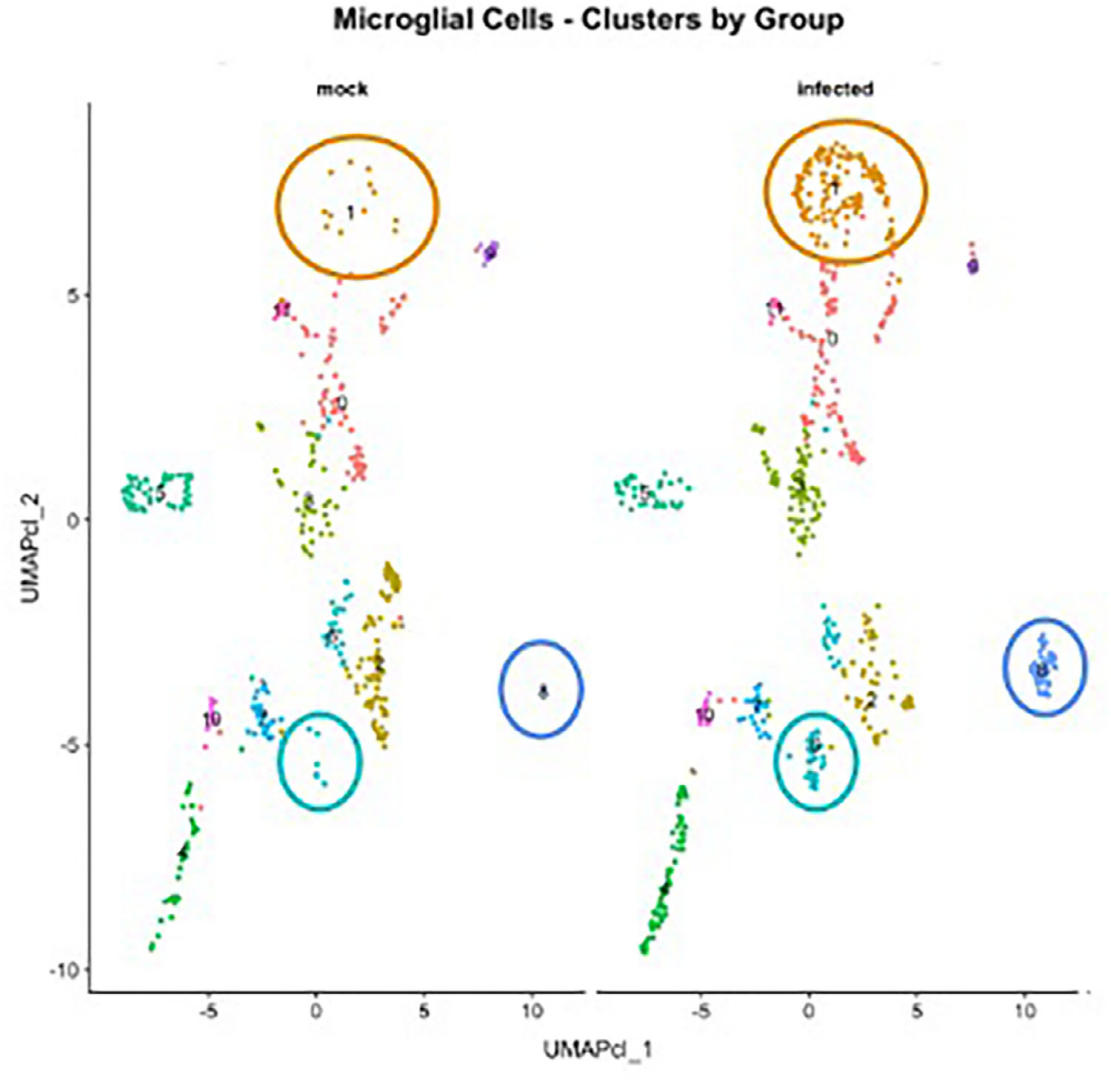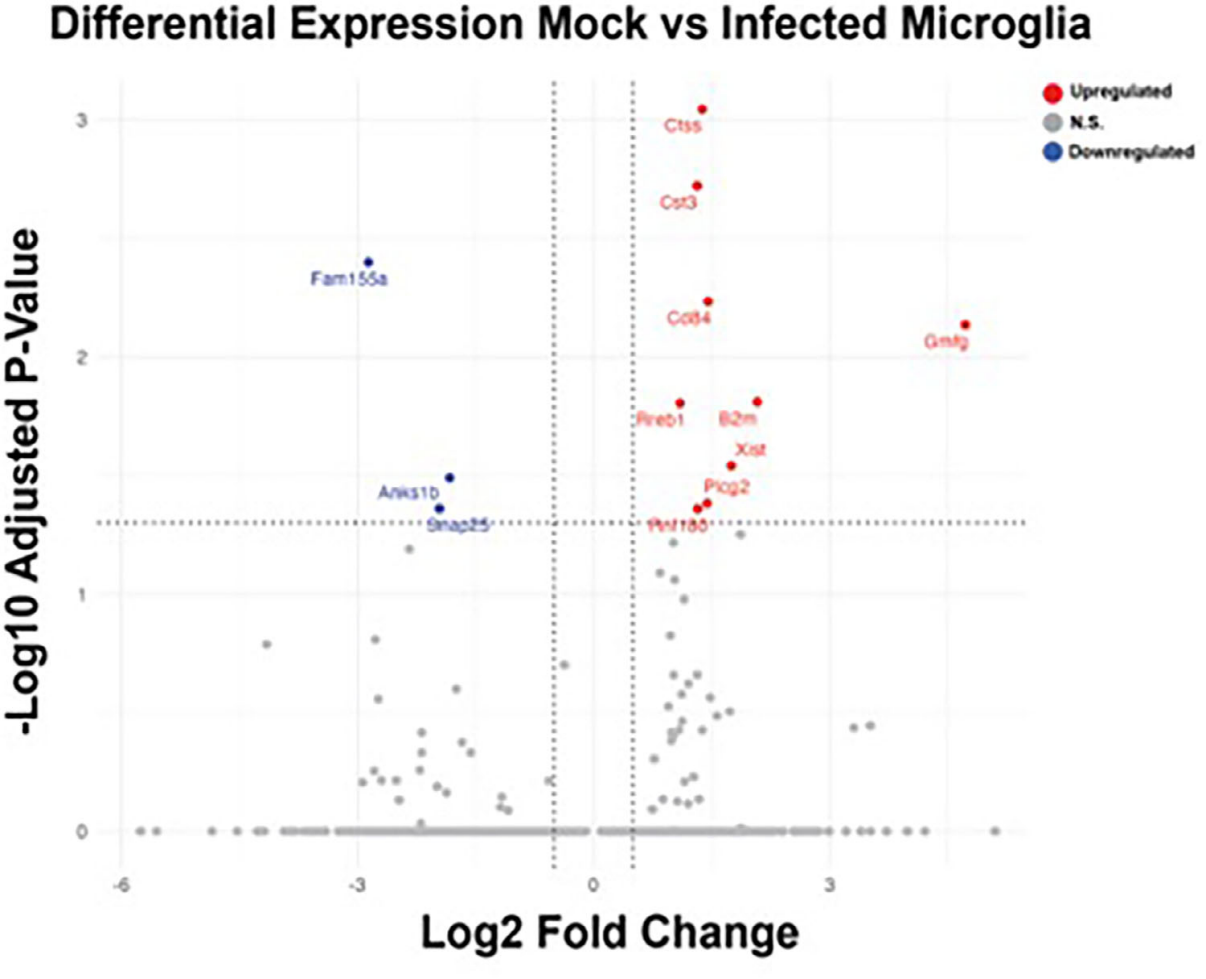# Multiomic Insights into Inflammation‐Driven Microglial Dysregulation in AD

**DOI:** 10.1002/alz70855_107308

**Published:** 2025-12-25

**Authors:** Stella R Wroblewski, Sara Morris, Gabrielle Blahusiak, Vivianne E Morrison, Elizabeth Engler‐Chiurazzi, Kevin Zwezdaryk

**Affiliations:** ^1^ Tulane University, New Orleans, LA, USA

## Abstract

**Background:**

Peripheral inflammation is linked to neurodegeneration. Using Xenium spatial transcriptomics (Figure 1), we test the hypothesis that peripheral inflammation triggers transcriptional changes in microglia. Preliminary data show microglial transcriptional changes are detectable within days of the peripheral inflammatory event, and moreover, that these changes persist for months. How this impacts Aβ and tau pathology is unknown.

**Method:**

3xTg‐AD mice were exposed to a peripheral (does not directly infect the CNS) viral infection inducing systemic inflammation. At 7 days‐ and 2‐months post infection, the right and left hemispheres of the brain, respectively, were harvested for immunostaining and integrated analysis of Multiome sequencing (simultaneous single‐cell RNA‐seq and ATAC‐seq) with spatial transcriptomics. Circulating cytokine levels in the blood were measured via ELISA.

**Result:**

At the 7‐day timepoint, spatial transcriptomics revealed 265 differentially expressed genes in microglia, with three unique microglial profiles present only in infected animals (Figure 2). Cluster features, such as downregulated Plcxd2 and Bhlhe40, have been associated with intracellular Aβ and pTau, as well as dysregulated lipid processing, respectively. Further, algorithmic cell sorting revealed overlap of microglia with glutamatergic neurons and endothelial cells, suggesting increased microglial engulfment of these cell types. Preliminary scRNA‐seq data at 2‐months post‐infection reveals a pro‐inflammatory microglial phenotype (Figure 3), as shown by increases in Ctss and Cd84. Genes critical for synaptic function and neuronal support (Snap25, Anks1b, Fam155a) were significantly downregulated, suggesting that microglia‐neuron interactions are influenced by peripheral inflammation. These changes correlated with global increased expression of Apoe and Psen1&2. Together, these findings show that peripheral inflammatory events induce significant deviations in microglia homeostasis.

**Conclusion:**

Our preliminary findings suggest that viral‐induced peripheral inflammation alters microglia state and homeostasis in a 3xTg‐AD model. Despite the clearance of systemic inflammation, the transcriptional profile of microglia remains different in infected vs mock‐infected mice. The data suggest peripheral inflammatory stress events alter microglial transcriptional profiles and a “reset” to the original baseline profile is not immediate. The implications on neurodegeneration are being explored.